# Arc silence aggravates traumatic neuronal injury via mGluR1-mediated ER stress and necroptosis

**DOI:** 10.1038/s41419-019-2198-5

**Published:** 2020-01-02

**Authors:** Tao Chen, Jie Zhu, Yu-Hai Wang, Chun-Hua Hang

**Affiliations:** 1 Department of Neurosurgery, Drum Tower Hospital, Medical School of Nanjing University, Nanjing, Jiangsu 210000 China; 2 Department of Neurosurgery, The 904th Hospital of PLA, Medical School of Anhui Medical University, Wuxi, Jiangsu 214044 China

**Keywords:** Cell death in the nervous system, Trauma

## Abstract

Delayed neuronal death is associated with neurological deficits and mortality after traumatic brain injury (TBI), where post-synaptic density (PSD) proteins are thought to play key roles. The immediate-early gene (IEG) coded protein Arc is a brain-specific PSD protein that controls synaptic plasticity and learning behaviors. In this study, we investigated the expression and biological function of Arc in neuronal death after TBI in an in vitro model mimicked by traumatic neuronal injury (TNI) in cortical neurons. TNI caused a temporal increase of Arc expression at 3 and 6 h. Knockdown of Arc expression using small interfering RNA (Si-Arc-3) promoted TNI-induced cytotoxicity and apoptosis. The results of western blot showed that Si-Arc-3 transfection further enhanced the activation of endoplasmic reticulum (ER) stress-associated factors, including glucose-regulated protein 78 (GRP78), C/EBP homologous protein (CHOP) and caspase-12 after TNI. In addition, knockdown of Arc significantly increased expression of (receptor-interacting protein kinase 1) RIP1 and the number of necroptotic cells, which were apparently prevented by necrostatin-1 (Nec-1). The results of immunostaining and western blot showed that knockdown of Arc activated the metabotropic glutamate receptor 1 (mGluR1) and intracellular Ca^2+^ release in neurons. Mechanistically, the Si-Arc-3-induced activation of ER stress-associated factors, RIP1 expression, apoptosis, and necroptosis were partially reversed by the mGluR1 antagonist AIDA. In summary, our data suggest that silence of Arc expression aggravates neuronal death after TNI by promoting apoptosis and necroptosis. These data support for the first time that Arc may represent a novel candidate for therapies against TBI.

## Introduction

Traumatic brain injury (TBI) is still a major public health problem worldwide, especially in China, where >10 million people suffered from TBI each year, mostly caused by road traffic incidents^1^. Owing to the advanced prevention strategy, enhanced monitoring, and care technology, and progressive neurosurgical training programs, outcomes after TBI have been substantially improved in the past few decades^2^. However, the mechanisms underlying neuronal injury after TBI are not well understood, and there is no effective treatment to date.

The major cause of neurological deficits and mortality after TBI is neuronal cell death, which occurs in two distinct ways, known as the primary and secondary death. The primary neuronal death represents a passive process with morphological features of disrupted membrane and organelle swelling and the secondary one refers to a delayed, programmed, and energy-dependent fashion characterized by nuclear condensation and fragmentation^3,4^. The TBI-induced neuronal death has been commonly delineated into two different categories: necrosis in the primary phase and apoptosis in the secondary phase. There are many forms of apoptosis, such as intrinsic apoptosis, extrinsic apoptosis, mitochondrial apoptosis, endoplasmic reticulum (ER)-associated apoptosis, and the iron-related apoptosis (ferroptosis), all of which can be observed be used as therapeutic target after TBI^5,6^. In addition, necroptosis, a recently discovered form of necrosis, is demonstrated to be a programmed cell death and participates in secondary neuronal cell death after TBI^7^. After decades of research, scientists believe that the optimal therapeutic strategy to limit TBI-induced neuronal death is the use of combined treatments that target multiple cell death pathways.

Immediate-early genes (IEGs) refer to a group of inducible genes with rapid activation kinetics after stimulation. Detection of IEG expression provides information regarding synaptic plasticity and cognitive function, and some IEG-coded proteins were demonstrated to play important roles in neurological dysfunctions^8^. The activity-regulated cytoskeletal (*Arc*), also known as *Arg3.1*, is a brain-specific IEG, and its transcriptional product Arc is a neuronal protein that is selectively expressed in post-synaptic density (PSD)^9^. In response to various neuronal activity, such as neurotransmitter release, *Arc* mRNA is rapidly induced and transferred to dendrites, where it translated into Arc protein and targeted to polysomes to regulate different forms of synaptic plasticity, such as long-term potentiation, long-term depression and homeostatic plasticity^10^. Previous studies showed that the expression of Arc was highly dynamic, and dysregulation of Arc and related signaling was associated with cognitive disorders, including autism and Alzheimer’s disease^11^. Our previous data showed that glutamate induced rapid induction of Arc via the NMDA receptor-mediated phosphorylation of ERK and CREB^12^. However, the role of Arc in neuronal injury after TBI has not been determined. In this study, we investigated the expression and biological function of Arc in traumatic neuronal injury (TNI) in cortical neurons.

## Materials and methods

### Primary culture of cortical neurons and TNI model

All animal research was approved by Nanjing University Committee on Animal Research. Cortical neurons were obtained from Sprague–Dawley rats at embryonic day 16–18 as previously described^13^. In brief, cerebral cortex was dissected and minced in Dulbelcco’s modified Eagle medium with l-glutamine plus 10% fetal bovine serum at 4 °C. Tissues were dissociated by 0.25% trypsin digestion for 15 min at 37 °C and gentle trituration. Then, neurons were resuspended in Neurobasal medium (NBM, Invitrogen, Carlsbad, CA, USA) containing 2% B27 supplement (B27, Invitrogen) and seeded in poly-d-lysine (50 μg/mL, 70–150 K, Sigma, St. Louis, MO, USA) precoated culture vessels. Neuronal cultures were maintained at 37 °C in a humidified incubator (5% CO_2_, 95% air, 98% humidity) and the culture medium was changed every other day. Cultures were utilized for in vitro experiments at 14–16 days when >95% of the cells were demonstrated to be neurons. The TNI model was performed according to our previously published method^14^. In brief, traumatic injury was performed on cultured neurons by using a rotating scribe injury device, which consisted of a rotating cylinder with 10 holes, steel needles, and a permanent magnet. The cylinder holes are distributed at the same interval from the center, and these holes allowed the 10 steel needles to freely cross through. A magnet is placed under the culture dish, which ensured that the steel needles could cling to the cell layer as the cylinder rotated. After one turn of this device, 10 concentric circular scratches were produced in the neuronal layer with equal distances (1.5 mm) between the scratches. This model, and its variants, are highly reproducible and are able to induce severe TNI.

### siRNA sequence and transfection

To knockdown the expression of Arc, three Arc targeted small interfering RNAs (siRNAs) and one control siRNA were synthesized by JiKai (Shanghai, China): Si-Arc-1 Sense: GCUGUCCCAGAUC -CAGAAU; Antisense: AUUCUGGAUCUGGGACAGC; Si-Arc-2 Sense: CCCAGAUCCAGAA -UCACAU; Antisense: AUGUGAUUCUGGAUCUGGG; Si-Arc-3 Sense: CCAACGUGAUC -CUGCAGAU; Antisense: AUCUGCAGGAUCACGUUGG Si-Control Sense: CCCUACCG -AAACUAAGCAU; Antisense: AUGCUUAGUUUCGGUAGGG;. These siRNA molecules were transfected using Lipofectamine RNAiMax reagent (Invitrogen) in Opti-MEM medium according to the manufacturer's instructions. After incubation for 48 h, culture media was changed to NBM containing 2% B27 supplement, and neurons were treated with TNI.

### Measurement of cell viability

Cell viability was measured by the WST-1 method using a kit according to the manufacture’s protocol (Jianchen bioengineer institute, Nanjing, Jiangsu, China). The results were expressed as a percentage of the control value.

### LDH release

The neurotoxicity in vitro was determined by measuring lactate dehydrogenase (LDH) release using a kit according to the manufacture’s protocol (Jiancheng bioengineer institute, Nanjing, Jiangsu, China).

### Measurement of apoptosis and necroptosis

Apoptosis in neurons were determined by measuring DNA fragmentation using a standard terminal deoxynucleotidyl transferase dUTP nick end labeling (TUNEL) staining method according to the manufacturer’s protocol (Roche, Penzberg, Germany). Double staining with propidium (PI, 10 μg/ml) and 4,6-diamidino-2-phenylindole (DAPI, 10 μg/ml) was used to detect necroptosis in neurons.

### Drugs and treatments

Salubrinal (SAL, SML0951) was purchased from Sigma-Aldrich Corporation (St. Louis, MO, USA). AEBSF (AEB, #78431) was obtained from Thermo Fisher Scientific (Waltham, MA, USA). Necrostatin-1 (Nec-1) and its “inactive” analog necrostatin-1i (Nec-1i) were purchased from Calbiochem (SanDiego, CA, USA). The metabotropic glutamate receptor 1 (mGluR1) antagonist 1-aminoindane-1,5,-dicarboxylic acid (AIDA, A254) and the mGluR5 antagonist 2-methyl-6-(phenylethynyl)-pyridine (MPEP, M5435) were obtained from Sigma (St. Louis, MO, USA). To investigate the role of ER stress, necroptosis or group I mGluRs in our experiments, 25 μM SAL, 300 μM AEB, 100 μM Nec-1, 100 μM Nec-1i, 100 μM AIDA or 5 μM MPEP was used immediately after TNI.

### Electron microscopy

The neurons were fixed with 2.5% glutaraldehyde in 0.1 M phosphate-buffered saline (PBS) at pH 7.4, post-fixed in 1% osmium tetroxide PBS, and embedded. Ultrathin sections were double stained with uranyl acetate and lead citrate and observed under an electron microscope (H-7500; Hitachi, Tokyo, Japan).

### Immunostaining

The neurons seeded on coverslips were fixed with 4% paraformaldehyde in PBS, treated with 0.1% Triton X-100, and then were blocked by 5% bovine serum albumin. The samples were incubated with the mGluR1 primary antibody (#12551, Cell Signaling) at 4 °C overnight. After being washed by PBS with Tween-20 (PBST) for three times, the samples were incubated with the secondary antibody at 37 °C for 1 h. Then, DAPI (10 μg/ml) was used to stain the nuclei, and the pictures were obtained using a Zeiss fluorescent imaging microscope (Carl Zeiss, Thornwood, NY, USA).

### Ca^2+^ imaging

Ca^2+^ imaging was performed using the Ca^2+^ indicator Fura-2 AM to measure the intracellular Ca^2+^ concentrations. The neurons cultured in coverslips were loaded with 5 μM Fura-2 AM in HBSS solution for 30 min and equilibrated lucifugally for 30 min. Cells were excited at 345 and 385 nm using a confocal laser scanning microscope, and the emission fluorescence at 510 nm was recorded. The fluorescence values were then plotted against time and shown as F/F_0_.

### Western blot analysis

A standard western blot assay was performed using the following primary antibodies: Arc (sc-17839, Santa Cruz, 1:300), GRP78 (#3183, Cell Signaling, 1:800); CHOP (#5554, Cell Signaling, 1:1000), cleaved-caspase-12 (#2202, Cell Signaling, 1:200), RIP1 (ab42126, Abcam, 1:1000), mGluR1 (#12551, Cell Signaling, 1:1000) and β-actin (ab8226, Abcam, 1:2000). After incubation with secondary antibodies for 1 h, the bands were visualized by using chemiluminescent detection system.

### Statistical analysis

Data represent the mean and standard error of the mean (SEM). Student’s *t* test (one-tailed for western blot and ratio quantification, two-tailed for the others) was performed for all statistical significance analysis using GraphPad Prism 6.0 software. All experiments were repeated at least for three times, and the number of repetition was indicated in the legends of each graph.

## Results

### Expression of Arc following TNI in cortical neurons

**Fig. 1:**
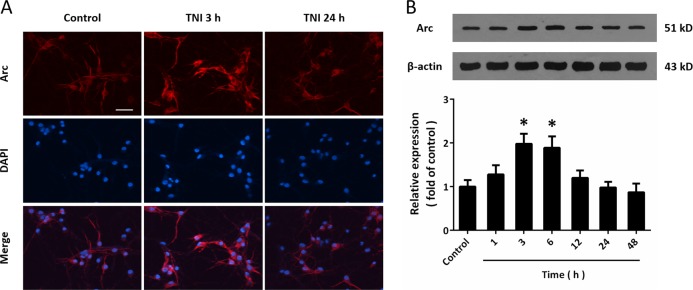
Expression of Arc following TNI in cortical neurons. **a** Immunocytochemistry shows that TNI upregulated Arc expression at 3 h, but not at 24 h in cortical neurons. **b** Western blot shows that TNI increased Arc expression at 3 and 6 h. Scale bar, 50 μm. Error bars indicate SEM (*n* = 6). *p < 0.05 vs. control group.

To mimic neuronal injury following TBI in vitro, cultured cortical neurons were treated with TNI, and immunocytochemistry was performed using Arc antibody and DAPI (Fig. 1a). The results showed that the intensity of Arc signal (red) in 3 h after TNI, but not 24 h after TNI, was higher than that in control neurons. However, the distribution of Arc in neurons was unaffected by TNI. In addition, the results of western blot showed that TNI caused a temporal increase of Arc expression at 3 and 6 h (Fig. 1b).

### Downregulation of Arc expression by siRNA transfection

**Fig. 2:**
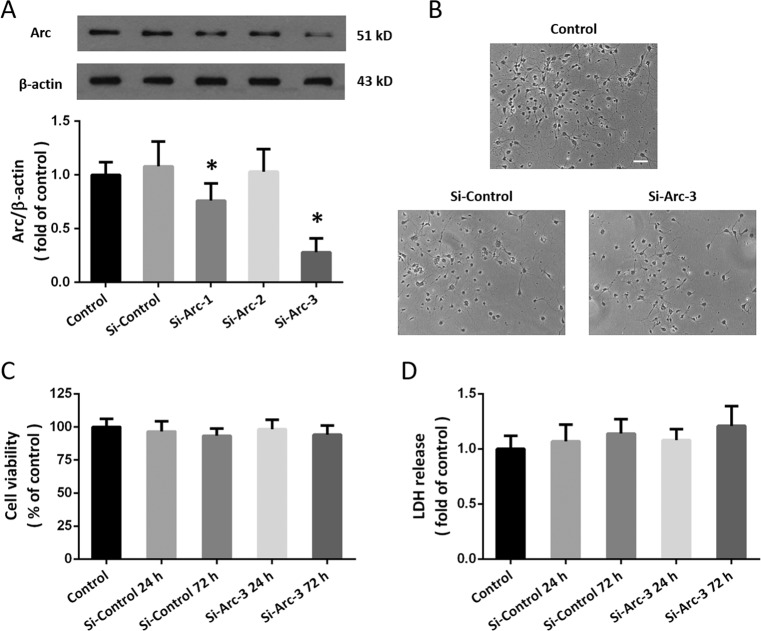
Downregulation of Arc expression by siRNA transfection. **a** Western blot shows that Si-Arc-1 and Si-Arc-3 significantly decreased Arc expression in cortical neurons. **b** No morphological changes were observed at 24 h after siRNA transfection. **c** Cell viability assay shows that siRNA transfection had no effect on cell viability. **d** LDH release assay shows that siRNA transfection had no effect on LDH release. Scale bar, 50 μm. Error bars indicate SEM (*n* = 6). *p < 0.05 vs. Si-control group.

To investigate the biological function of Arc in TNI, we designed three specific targeted siRNAs (Si-Arc-1, Si-Arc-2, and Si-Arc-3), which were transfected to neurons with the control siRNA (Si-Control). The results showed that Si-Arc-1 and Si-Arc-3, but not Si-Arc-2, significantly reduced Arc expression in neurons (Fig. 2a). Si-Arc-3 was used in following experiments because of its higher knockdown efficiency. As shown in Fig. 2b, transfection with Si-Arc-3 or Si-Control for 72 h did not change the morphology of neurons. The results of cell viability (Fig. 2c) and LDH release (Fig. 2d) showed that transfection with these siRNAs had no obvious cytotoxic effect on cortical neurons.

### Knockdown of Arc aggravates TNI-induced cytotoxicity

**Fig. 3:**
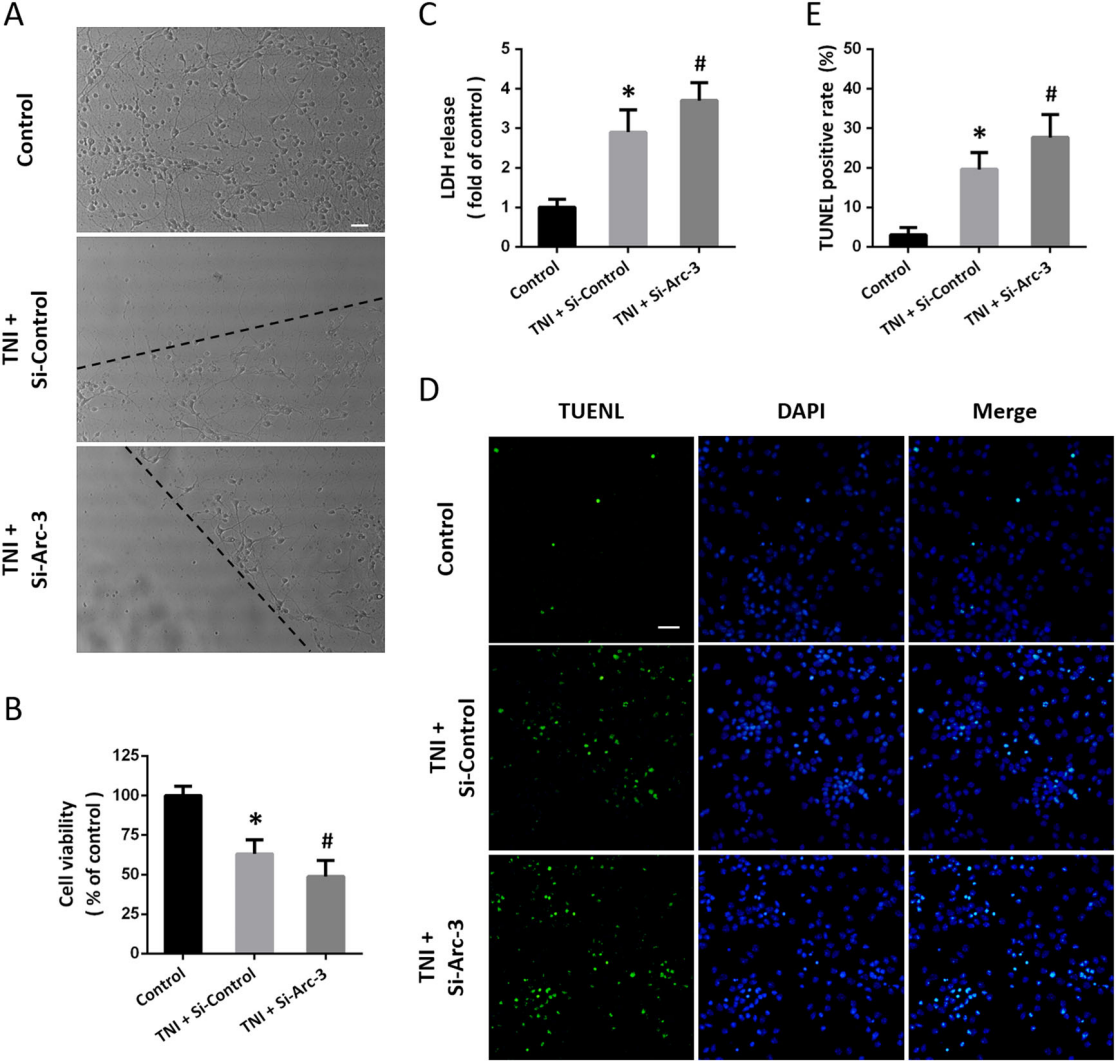
Knockdown of Arc aggravates TNI-induced cytotoxicity. **a** Morphological changes after Arc knockdown and TNI are shown. **b** Cell viability assay shows that knockdown of Arc further decreased cell viability after TNI. **c** LDH release assay shows that knockdown of Arc further increased LDH release after TNI. **d**, **e** TUNEL staining **d** and quantification **e** show that knockdown of Arc further increased neuronal apoptosis after TNI. The dotted line indicates the edge of the injury. Scale bar, 50 μm. Error bars indicate SEM (*n* = 6). *p < 0.05 vs. Control group. ^#^*p* < 0.05 vs^.^ TNI + Si-Control group.

After TNI, neurons with damaged neurites and shrunken cell bodies were observed, which was aggravated by transfection with Si-Arc-3 (Fig. 3a). The cell viability in Si-Arc-3 transfected and TNI-injured neurons was lower than that in Si-Control transfected and TNI-injured neurons (Fig. 3b). The increased LDH release after TNI was enlarged by Arc knockdown (Fig. 3c). We also detected neuronal apoptosis using TUNEL staining (Fig. 3d), and the results showed that TNI-induced apoptosis was increased by Si-Arc-3 transfection (Fig. 3e).

### Knockdown of Arc promotes ER stress after TNI

**Fig. 4:**
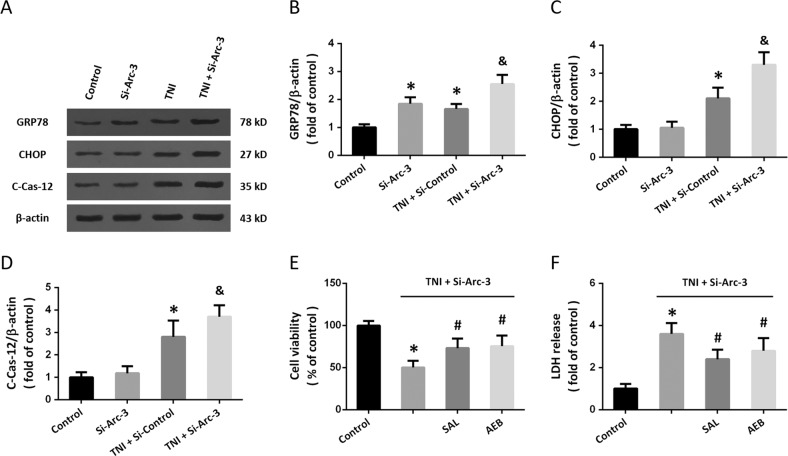
Knockdown of Arc promotes ER stress after TNI. **a**–**d** Western blot **a** and quantification **b**–**d** show that knockdown of Arc further increased the expression of GRP78 **b**, CHOP **c**, and Caspase-12 **d** after TNI. **e** Cell viability assay shows that SAL and AEB partially prevented the Si-Arc-3-induced decrease of cell viability after TNI. **f** LDH release assay shows that SAL and AEB partially prevented the Si-Arc-3-induced increase of LDH release after TNI. Error bars indicate SEM (*n* = 6). *p < 0.05 vs. control group. ^&^*p* < 0.05 vs^.^ TNI + Si-Control group. ^#^*p* < 0.05 vs. TNI + Si^-^Arc-3 group.

To investigate the effect of Arc on ER stress after TNI, the expression of ER stress-associated proteins was detected by western blot (Fig. 4a). Knockdown of Arc significantly increased the expression of GRP78 both in the presence and absence of TNI in cortical neurons (Fig. 4b). Transfection with Si-Arc-3 had no effect on CHOP and cleaved-caspase-12 but increased their expression after TNI (Figs. 4c–e). To confirm the involvement of ER stress, cortical neurons were pretreated with the ER stress inhibitor salubrinal (SAL, 25 μM) or AEBSF (AEB, 300 μM). The results of cell viability (Fig. 4e) and LDH release (Fig. 4f) showed that the cytotoxicity induced by TNI and Arc knockdown was partially prevented by both SAL and AEB.

### Knockdown of Arc augments necroptosis after TNI

**Fig. 5:**
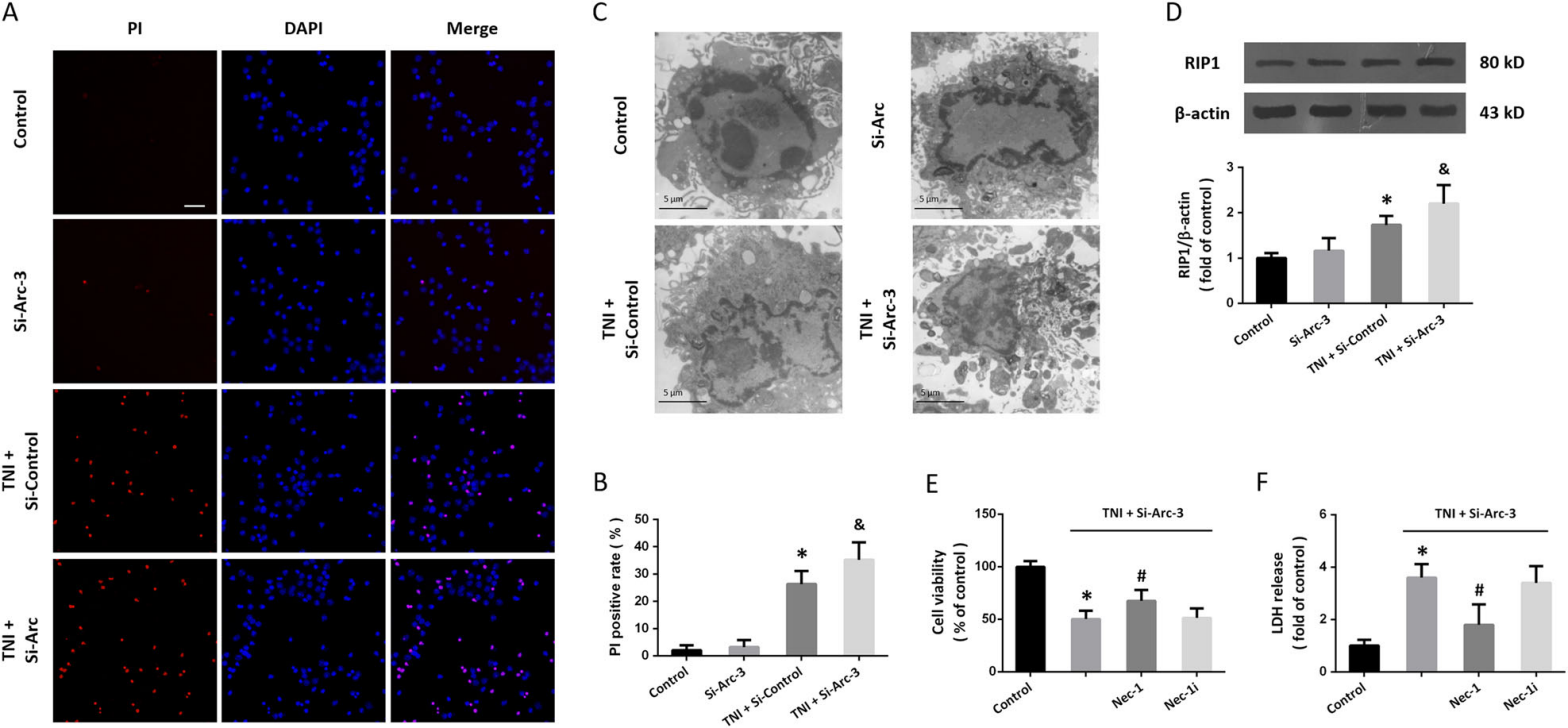
Knockdown of Arc augments necroptosis after TNI. **a**, **b** PI staining **a** and quantification **b** show that knockdown of Arc further increased neuronal necrosis after TNI. **c** Electron microscopy shows morphological changes of necroptosis in neurons after TNI and Si-Arc-3 transfection. **d** Western blot shows that knockdown of Arc further increased RIP1 expression after TNI. **e** Cell viability assay shows that Nec-1, but not Nec-1i, partially prevented the Si-Arc-3-induced decrease of cell viability after TNI. **f** LDH release assay shows that Nec-1, but not Nec-1i, partially prevented the Si-Arc-3-induced increase of LDH release after TNI. Error bars indicate SEM (*n* = 6). *p < 0.05 vs. control group. ^&^*p* < 0.05 vs^.^ TNI + Si-control group. ^#^*p* < 0.05 vs. TNI + Si^-^Arc-3 group.

Next, we performed PI staining to detect necrotic cell death in cortical neurons (Fig. 5a). The results showed that transfection with Si-Arc-3 had no effect on necrosis, but it significantly increased the number of PI-positive cells in TNI-treated neurons (Fig. 5b). As shown in Fig. 5c, some of the neurons treated with TNI and Si-Arc-3 displayed loss of plasma membrane integrity and disintegration of organelles, the classic morphology of necrosis. In addition, the results of western blot showed that downregulation of Arc increased the expression of RIP1 after TNI, indicating the involvement of necroptosis (Fig. 5d). The Si-Arc-3-induced aggravation of cytotoxicity after TNI, as measured by cell viability (Fig. 5e) and LDH release (Fig. 5f), was partially prevented by the necroptosis inhibitor Nec-1, but not by its dysfunctional analog Nec-1i.

### Knockdown of Arc activates mGluR1 signaling after TNI

**Fig. 6:**
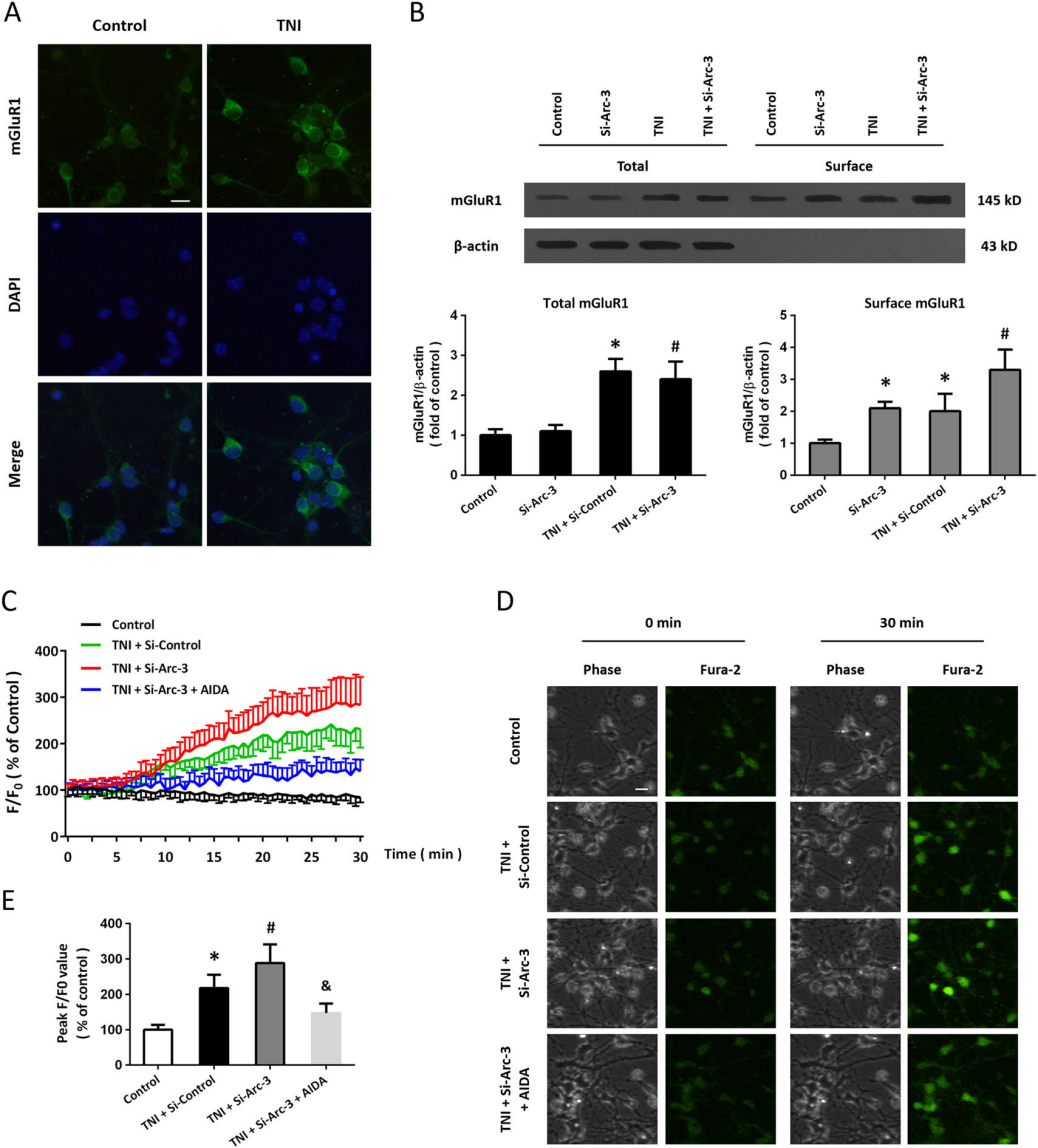
Knockdown of Arc activates mGluR1 signaling after TNI. **a** Immunocytochemistry shows that TNI upregulated mGluR1 expression in cortical neurons. **b** Western blot shows that knockdown of Arc further increased the total and surface expression of mGluR1 after TNI in cortical neurons. **c**–**e** Ca^2+^ imaging **c**, **d** and quantification **e** shows that knockdown of Arc further increased intracellular Ca^2+^ concentration induced by TNI, which was attenuated by the mGluR1 antagonist AIDA. Error bars indicate SEM (*n* = 6). *p < 0.05 vs. control group. ^#^*p* < 0.05 vs^.^ TNI + Si-Control group. ^&^*p* < 0.05 vs. TNI + Si^-^Arc-3 group.

We performed immunocytochemistry using the mGluR1 antibody (Fig. 5a), and the results showed that TNI increased the immunofluorescence intensity of mGluR1 in cortical neurons. In congruent, the results of western blot showed that TNI increased the total protein levels of mGluR1, but Si-Arc-3 had no effect on total mGluR1 expression (Fig. 6b). However, the surface expression of mGluR1 in cortical neurons were significantly increased by both Si-Arc-3 and TNI (Fig. 6b). Next, we performed Ca^2+^ imaging in Ca^2+^-free solution to examine the role of mGluR1 in our in vitro model (Fig. 6c). The representative pictures of Ca^2+^ imaging were shown as Fig. 6d. The results showed that TNI led to an increase in intracellular Ca^2+^ concentration, which was further increased by Arc knockdown. The intracellular Ca^2+^ release induced by TNI and Si-Arc-3 was markedly alleviated by the mGluR1 antagonist AIDA (Fig. 6e).

### Role of mGluR1 pathway in Arc knockdown-induced ER stress and necroptosis

**Fig. 7:**
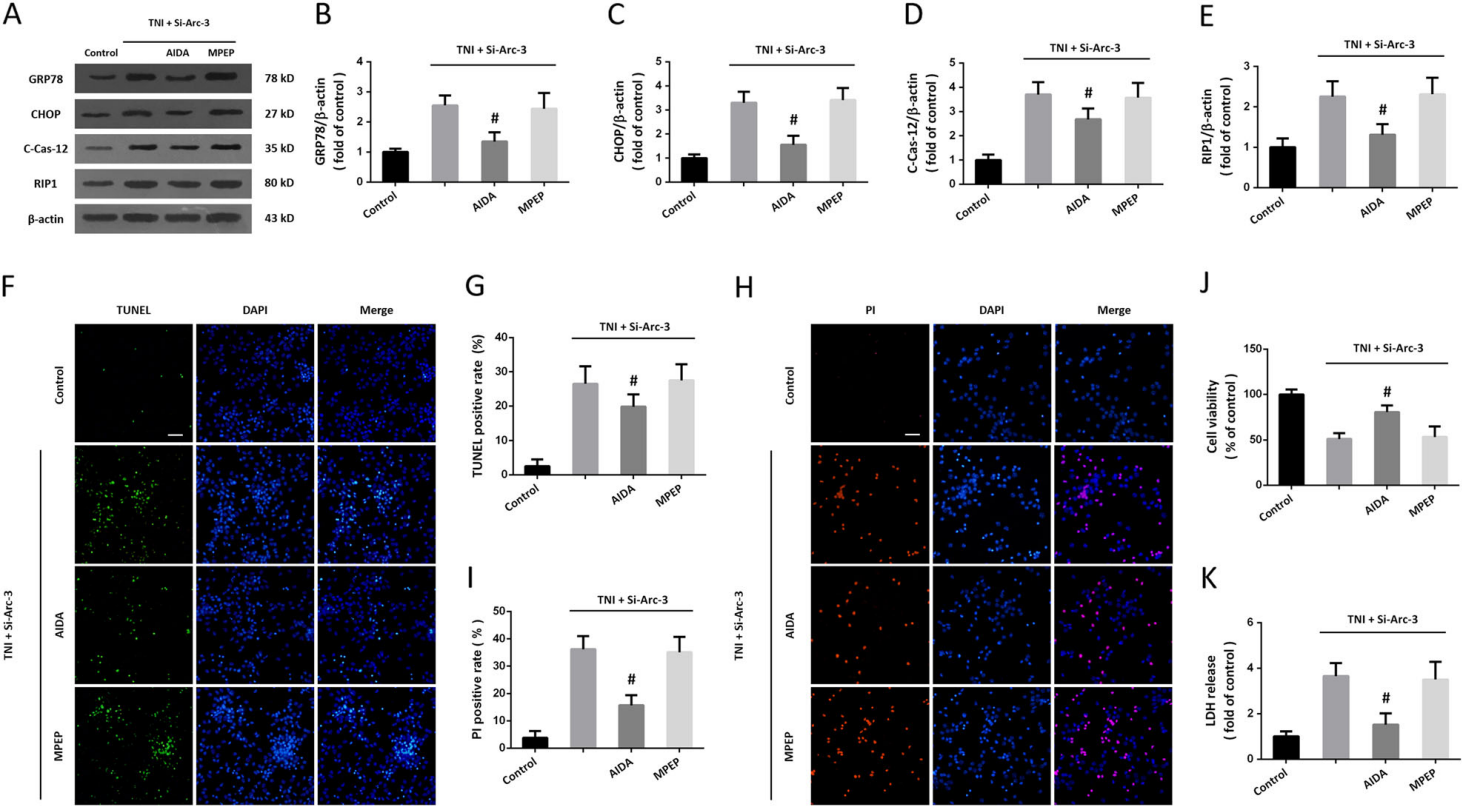
Role of mGluR1 pathway in Arc knockdown-induced ER stress and necroptosis. **a**–**e** Western blot **a** and quantification **b**–**e** show that the increased expression of GRP78 **b**, CHOP **c**, cleaved-caspase-12 **d**, and RIP1 **e** induced by Arc knockdown after TNI were partially prevented by AIDA, but not by MPEP. **f**, **g** TUNEL staining **f** and quantification **g** show that the enhanced neuronal apoptosis induced by Arc knockdown after TNI was attenuated by AIDA, but not by MPEP. **h**, **i** PI staining **h** and quantification **i** show that the increased number of PI-positive cells induced by Arc knockdown after TNI was inhibited by AIDA, but not by MPEP. **j** Cell viability assay shows that the decreased cell viability induced by Arc knockdown after TNI was alleviated by AIDA, but not by MPEP. **k** LDH release assay shows that the increased LDH release induced by Arc knockdown after TNI was partially prevented by AIDA, but not by MPEP. Error bars indicate SEM (*n* = 6). *p < 0.05 vs. Control group. ^#^*p* < 0.05 vs^.^ TNI + Si-Arc-3 group.

To further investigate the role of mGluR1 pathway in our study, we repeated the above experiments using the mGluR1 antagonist AIDA and the mGluR5 antagonist MPEP. The expression of ER stress-associated proteins and RIP1 were detected by western blot (Fig. 7a). The results showed that the increased expression of GRP78 (Fig. 7b), CHOP (Fig. 7c), cleaved-caspase-12 (Fig. 7d), and RIP1 (Fig. 7e) induced by TNI and Si-Arc-3 were inhibited by AIDA but not altered by MPEP. The neuronal apoptosis and necroptosis were detected by TUNEL staining (Fig. 7f) and PI (Fig. 7h) staining, respectively. We found that AIDA, not MPEP, decreased both apoptosis (Fig. 7g) and necroptosis (Fig. 7i) in cortical neurons after TNI and Arc knockdown. As shown in Fig. 7j, k, similar results of cell viability and LDH release were also observed.

## Discussion

**Fig. 8:**
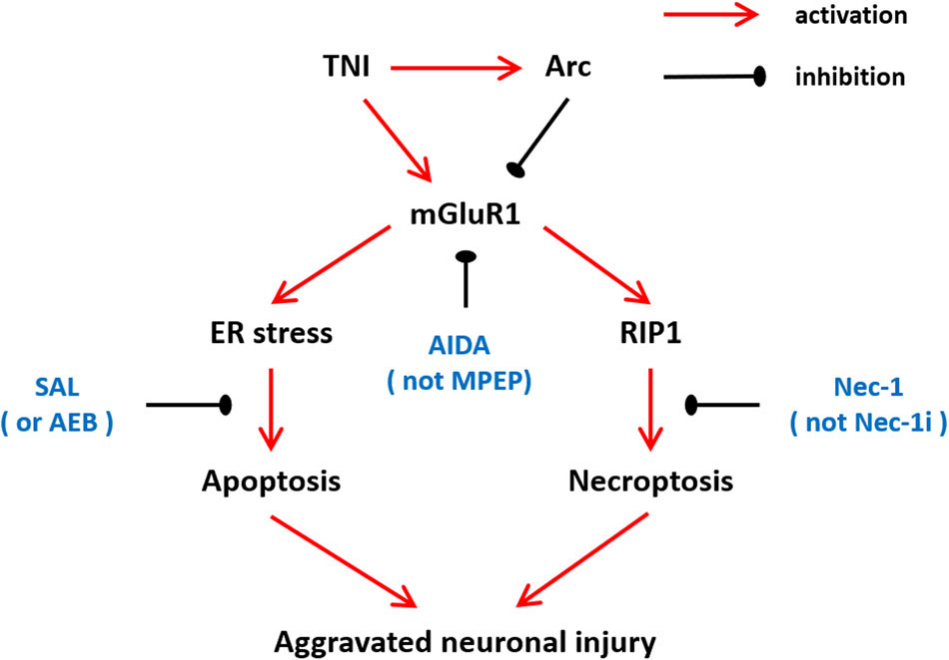
A proposed diagram tying together the observations involved in the mGluR1-mediated regulation of ER stress and necroptosis after Arc knockdown and TNI in cortical neurons.

The molecular mechanisms underlying TNI are complicated, and to date, there is no drug or treatment that can effectively attenuate neuronal death after TBI. Identification of new drug targets for TBI is urgently needed for the development of new therapies, and many recent data show that PSD proteins might be ideal candidates^15^. In this study, we identified Arc as an endogenous factor that inhibit neuronal apoptosis and necroptosis after TNI via regulation of mGluR1 signaling (Fig. 8). We found that (a) TNI causes a temporal increase of Arc expression in vitro; (b) knockdown of Arc via siRNA transfection has no cytotoxic effect in neurons; (c) knockdown of Arc aggravates the TNI-induced cytotoxicity; (d) knockdown of Arc promotes ER stress-related apoptosis and necroptosis after TNI; and (e) knockdown of Arc activates mGluR1 and intracellular Ca^2+^ release after TNI.

As an IEG whose induction is strongly correlated with synaptic plasticity and cognitive function, Arc is dynamically regulated by complicated mechanisms at both transcription and translation levels. Many previous studies focused on the regulation of Arc mRNA, which was found to be associated with Ca^2+^, cAMP, protein kinase (PKA), protein kinase C (PKC), ERK, as well as various receptors, including dopamine, brain-derived neurotrophic factor (BDNF), insulin, and glutamate receptors^16^. At post-transcriptional level, the Arc protein synthesis was increased by BDNF and reelin-integrin receptor in synaptoneurosome preparations^17,18^. Previous studies showed that Arc translation was controlled by convergent NMDA and G_s_-coupled receptor signaling pathways^19^. Our previous study showed that glutamate induced rapid induction of Arc via the NMDA receptor-mediated phosphorylation of ERK and CREB^12^, indicating the potential involvement of Arc dysregulation in neurological disorders. Congruently, elevated Arc protein levels have been found in Fragile X Syndrome, Angelman Syndrome (AS) and Alzheimer’s disease (AD)^11^. In the present study, expression of Arc expression was determined in cultured cortical neurons using fluorescence staining and western blot. A transitory increase of Arc protein level was found after TNI, indicating the dysregulation of neuronal injury Arc in TBI. Whether some molecular mechanisms with protein degradation are involved needs to be determined in future.

The important role of IEG-coded proteins in cell death in the central nervous system (CNS) has been well studies for many years^20,21^. Recently, more and more attention was paid on the IEG-coded PSD proteins, many of which were found to be protective or detrimental in neurological disorders, including TBI^15,22,23^. Previous studies showed that Homer1a, an IEG-coded neuron specific PSD protein, protects neuronal cells against tumor necrosis factor-α/cycloheximide treatment, oxidative stress, as well as TBI-induced cell death^24–26^. Downregulation of another novel PSD protein Preso was found to reduce glutamate-induced excitotoxicity by differentially regulating glutamate receptors in neurons^27^. Our previous study showed that glutamate induced rapid induction of Arc via the NMDAR signaling, indicating the potential role of Arc in neuronal excitotoxicity. In addition, Arc was demonstrated to be negatively regulated by AMPAR via the pertussis toxin-sensitive G protein-dependent mechanism^28^. To determine the role of endogenous Arc in traumatic neuronal death, three different siRNA molecules were designed, and the Si-Arc-3 was used owing to its high efficiency. Silence of Arc expression via Si-Arc-3 transfection significantly aggravated TNI-induced cytotoxicity as evidenced by cell viability, LDH release, and TUNEL staining. These data suggest that the induction of Arc protein in neurons might be an endogenous protective mechanism after TBI.

Multiple previous studies have been shown that Arc has close relationship with glutamate receptor signaling. Activation of the group I mGluRs led to the increased expression of Arc protein in neuronal dendrites through translational mechanisms^29^, whereas in vivo treatment with the NMDAR antagonists during high frequency performant path stimulation was found to block the targeting of Arc to dendrites^30^. In contrary, Arc selectively regulated the trafficking of AMPAR by accelerating endocytosis and reducing surface expression in neurons^31^, indicating the cross-talk of Arc and glutamate receptors. Owing to the limited distribution and multiplex functions of mGluRs, especially group I mGluRs, they are considered to be ideal targets with less side-effects for TBI therapy^15^. Previous studies suggested that blocking the activation of group I mGluRs using selective antagonists exerted protective effects against excitotoxic neuronal death, which was found to be mainly dependent on the inhibition of mGluR1^32,33^. In congruent, the results of immunofluorescence using mGluR1 antibody showed that TNI significantly increased the expression of mGluR1 in cortical neurons. The results of western blot using surface biotinylation assay further showed that knockdown of Arc increased mGuR1 expression in TNI-injured neurons. As a member of class C G-protein-coupled receptor super family proteins, mGluR1 regulates the phospholipase C/inositol -1,4,5-triphosphate (IP_3_)/Ca^2+^ pathway to trigger intracellular signaling cascades^34^. Thus, we further measured intracellular Ca^2+^ metabolism using Ca^2+^ imaging in cortical neurons, and we found that the intracellular Ca^2+^ release induced by TNI and Si-Arc-3 was markedly alleviated by the mGluR1 antagonist AIDA. These data strongly indicate that downregulation of Arc activates the mGluR1 signaling to mediate the TNI-induced neuronal damage in vitro.

Perturbation in ER homeostasis, including protein folding, redox balance, and Ca^2+^ metabolism, may lead to the accumulation of misfolded and unfolded proteins in the ER lumen, a process named “ER stress”^35^. In response to ER stress, a short term adaptive mechanism referred as unfolded protein response is triggered to restore protein homeostasis by activating various intracellular signal transduction reactions^36^. Under pathological conditions, the prolonged or excessive ER stress results in the initiation of apoptosis through detrimental cascades, such as CHOP, caspase-12 and intracellular Ca^2+^ release. Increasing evidenced have shown that ER stress-related apoptosis is involved in neuronal loss of many neurological disorders, including TBI. Previous studies using TNI animal models showed that increased expression of CHOP and activation of caspase-12 were detected at frontal cortex after TBI^37,38^. Congruently, our present data showed that TNI significantly increased the expression of GRP78, CHOP and caspase-12 in cortical neurons. In addition, we observed the aggravation of intracellular Ca^2+^ release after Arc downregulation, which was mediated by the mGluR1 pathway. Thus, we further determined the potential involvement of ER stress in our in vitro model. Recently, the ER stress-associated apoptosis has been demonstrated to be therapeutic targets of many neuroprotective agents after TBI^39,40^. The results of western blot showed that knockdown of Arc provoked the activation of ER associated factors, and the effects of si-Arc on neuronal damage were partially prevented by ER stress inhibitors, indicating that ER stress-related apoptosis contributed to si-Arc induced aggravation of neuronal damage after TNI.

Necroptosis, a recently defined form of programmed necrosis, can be triggered by various stimuli from both intracellular and extracellular. Necroptotic cells display disrupted plasma membrane and cell lysis, and can be observed in a variety of cell types, including neurons^41,42^. Molecularly, necroptosis is not related to activation of caspase family proteins, but dependent on RIP1 signaling. Under stress conditions, dimerization of RIP1 results in the formation of the RIP1-RIP3-MLKL complex, known as complex IIb, which in turn leads to membrane disruption and cell lysis^43^. In this study, loss of plasma membrane integrity and disintegration of organelles were found in neurons after TNI and Arc knockdown, which was accompanied by increased number of PI-positive cells and enhanced expression of RIP1 protein. Thus, necroptotic cell death might contribute to TNI-induced cell death and Si-Arc-induced aggravation of neuronal damage. Inactivation of RIP1 using genetic mutations or pharmacological agents has been shown to be neuroprotective against neurological disorders^44,45^. For example, the RIP1 inhibitor nec-1 was found to reduce histopathology and improve functional outcome after controlled cortical impact^46^. Our data showed that neuronal toxicity after Arc knockdown was partially prevented by Nec-1 treatment, but not by its dysfunctional analog Nec-1i. Increasing evidence demonstrated that inhibition of RIP1-associated necroptosis was a protective mechanism that mediates therapeutic effects of many agents and strategies^7,47,48^. Intriguingly, we found that the increased neuronal injury and expression of RIP1 induced by TNI and Si-Arc-3 were inhibited by AIDA but not altered by MPEP, indicating that mGluR1 might be the upstream signal of RIP1-dependent necroptosis. However, to date, there are no reports about the regulation of mGluRs on RIP1 activation, the exact mechanism of which needs to be further determined.

In conclusion, Arc silencing aggravates neuronal injury induced by TNI by promoting ER stress-associated apoptosis and necroptosis in cortical neurons. The mechanisms underlying these effects involve in the activation of mGluR1-mediated ER stress and RIP1-dependent necroptosis. Thus, Arc might be an ideal therapeutic target for neuroprotective research against TBI.
